# Unexpected Identification of Two Medialized Tympanostomy Tubes in an Adult: A Case Report

**DOI:** 10.7759/cureus.116172

**Published:** 2026-09-13

**Authors:** Loga S Iyer, Anita Jeyakumar

**Affiliations:** 1 Otolaryngology, Northeast Ohio Medical University, Akron, USA; 2 Otolaryngology - Head and Neck Surgery, HEARS Otology, Akron, USA

**Keywords:** adult otology, eustachian tube dysfunction, medial displacement, otologic complications, tympanostomy tubes

## Abstract

Tympanostomy tube placement is one of the most commonly performed otologic procedures and is widely used in the management of chronic otitis media with effusion (OME), recurrent acute otitis media (AOM), and Eustachian tube dysfunction (ETD). Although generally safe, complications may occur, and medialization of a tympanostomy tube into the middle ear space is a rare but recognized complication that may be associated with surgical removal due to the potential for chronic inflammation, conductive hearing loss, or middle ear injury. We present a case of two medialized tympanostomy tubes identified intraoperatively in the middle ear of a 36-year-old woman with bilateral ETD and no prior history of childhood ear disease or otologic surgery. The patient underwent left exploratory tympanoplasty with removal of two medialized tubes and right tympanostomy tube removal with patch repair, with an uncomplicated postoperative course. The outside operative documentation reported placement of one left T-tube; therefore, the timing and mechanism by which the second tube became medialized could not be established. This case highlights the unusual finding of two medialized tympanostomy tubes in an adult patient and underscores the importance of recognizing this rare complication and considering surgical management when clinically indicated.

## Introduction

Tympanostomy tube placement is one of the most commonly performed otologic procedures and is widely used in the management of chronic otitis media with effusion (OME), recurrent acute otitis media (AOM), and Eustachian tube dysfunction (ETD). The procedure involves creating a myringotomy in the tympanic membrane and inserting a ventilation tube to facilitate middle ear aeration and prevent the accumulation of fluid.

Tympanostomy tubes may be categorized as short term or long term based on their intended duration of placement. Short-term tubes are designed to remain in the tympanic membrane for several months to approximately one year, whereas long-term tubes, such as T-tubes, are designed to provide prolonged middle ear ventilation and are often used in patients with persistent ETD. Although generally safe, tympanostomy tube placement is associated with complications including otorrhea, tympanosclerosis, persistent tympanic membrane perforation, and premature extrusion [1].

Medialization of a tympanostomy tube into the middle ear space is an uncommon complication of tube placement. In pediatric populations, reported rates range from approximately 0.5% to 1.1% [1,2]. These estimates are derived primarily from pediatric studies and should not be extrapolated to adults, in whom the incidence remains poorly established. Proposed mechanisms include displacement through the tympanic membrane during or after tube placement, enlargement of the myringotomy, and persistent negative middle ear pressure associated with ETD [2,3]. The available adult literature is limited largely to individual case reports.

We present an unusual case of two medialized tympanostomy tubes identified unexpectedly within the middle ear space of an adult patient with ETD who underwent surgical removal.

## Case presentation

A 36-year-old woman with a history of bilateral ETD was referred to our tertiary otology practice for evaluation of a presumed medially migrated tympanostomy tube in the left ear. The patient had previously undergone bilateral tympanostomy tube placement at an outside facility for ETD and recurrent otitis media. The available records did not specify which indication applied to each ear. She denied a history of childhood ear disease or other prior otologic surgery. Her otologic symptoms began at approximately 31 years of age and initially consisted of left-sided otalgia and aural fullness. A left tympanostomy tube was subsequently placed, followed by right tympanostomy tube placement after she developed similar symptoms in the right ear. During subsequent follow-up, the left tympanostomy tube was noted to have migrated medially into the middle ear space, prompting referral to our practice. Preoperative otoscopic examination demonstrated a medially displaced tympanostomy tube behind the left tympanic membrane; the presence of a second tube was not appreciated preoperatively. A preoperative audiogram obtained from the referring practice demonstrated only a minimal conductive component (Figure 1).

**Figure 1:**
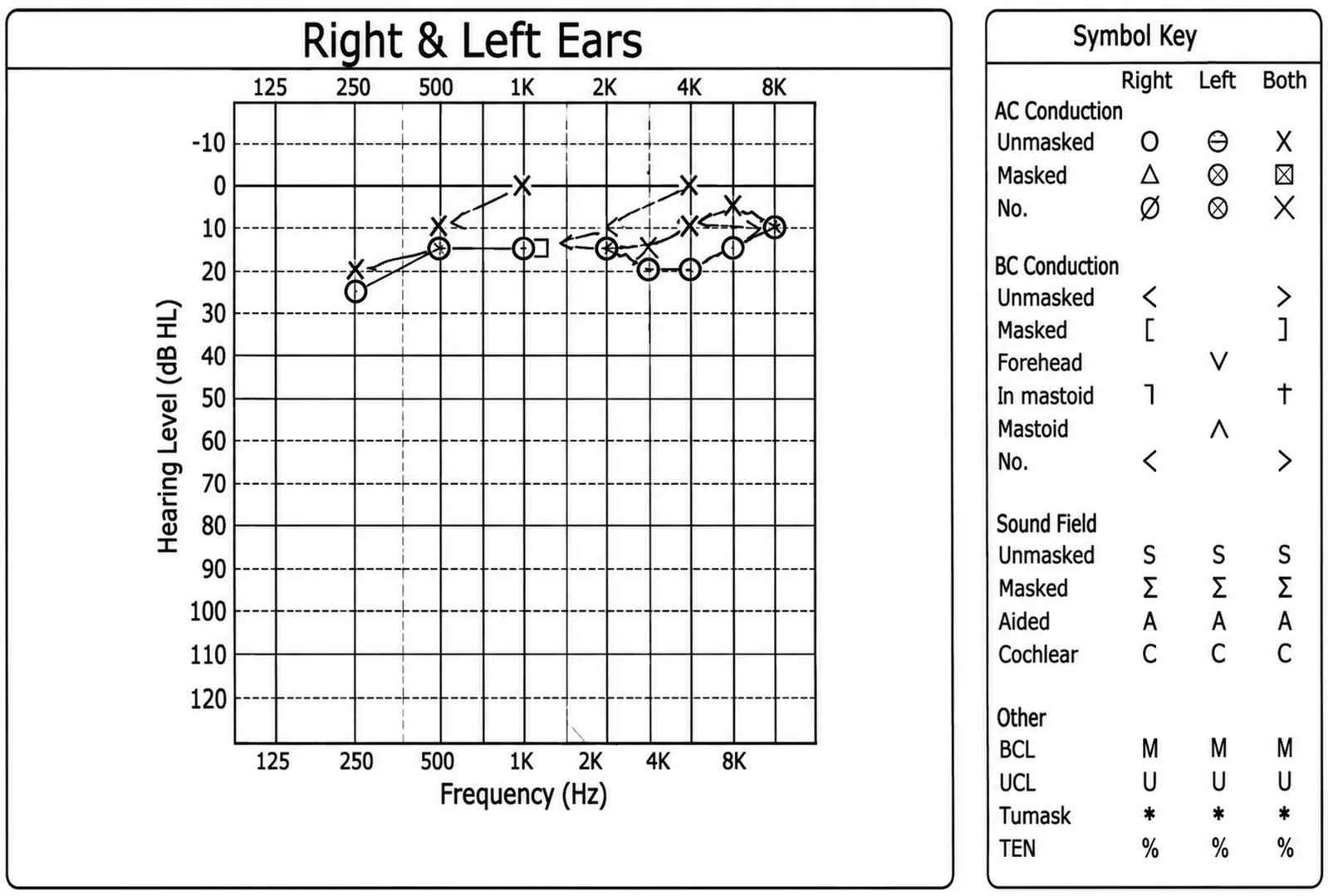
Preoperative pure-tone audiogram obtained from the referring practice. Hearing sensitivity was within normal limits bilaterally, with a small low-frequency air-bone gap in the right ear. Figure 1 is a preoperative audiogram that was provided by the outside practice as part of the patient's medical records.

The patient's medical history was also notable for mild seasonal allergic rhinitis, for which she had been using intranasal fluticasone. Given the potential contribution of allergic rhinitis to her ETD, she was treated with topical nasal steroid therapy. Following initiation of allergy treatment, she experienced significant improvement in her otalgia and stabilization of her ETD. Nasopharyngoscopy demonstrated normal nasopharyngeal anatomy without evidence of obstructive pathology. No temporal bone imaging was obtained because the preoperative clinical evaluation did not indicate a need for imaging. Preoperative tympanometry was not available in the referring records.

Because her otologic symptoms and recurrent ear infections had resolved following allergy treatment, the patient was observed for six months while continuing medical management. During this period, she remained free of otalgia and ear infections. Outside operative documentation reported placement of a single T-tube in the left ear, and the patient likewise recalled placement of only one left-sided T-tube. Therefore, the origin and timing of the second tube ultimately identified within the middle ear could not be established from the available records. It remains unclear whether the second tube represented a separately placed tube or another episode of medialization. This limitation prevents definitive characterization of the finding as recurrent or repeated medial migration.

Given the persistent presence of the medialized tube and the potential risks associated with a retained foreign body within the middle ear space, the decision was made to proceed with surgical removal. The patient underwent exploratory left tympanoplasty for removal of the presumed single medialized T-tube. Intraoperatively, two T-tubes were unexpectedly identified within the middle ear space and were subsequently removed. The right tympanostomy tube was also removed with patch repair of the tympanic membrane (Figure 2). No gross evidence of middle ear inflammation, ossicular injury, cholesteatoma, or other significant middle ear pathology was identified intraoperatively.

**Figure 2:**
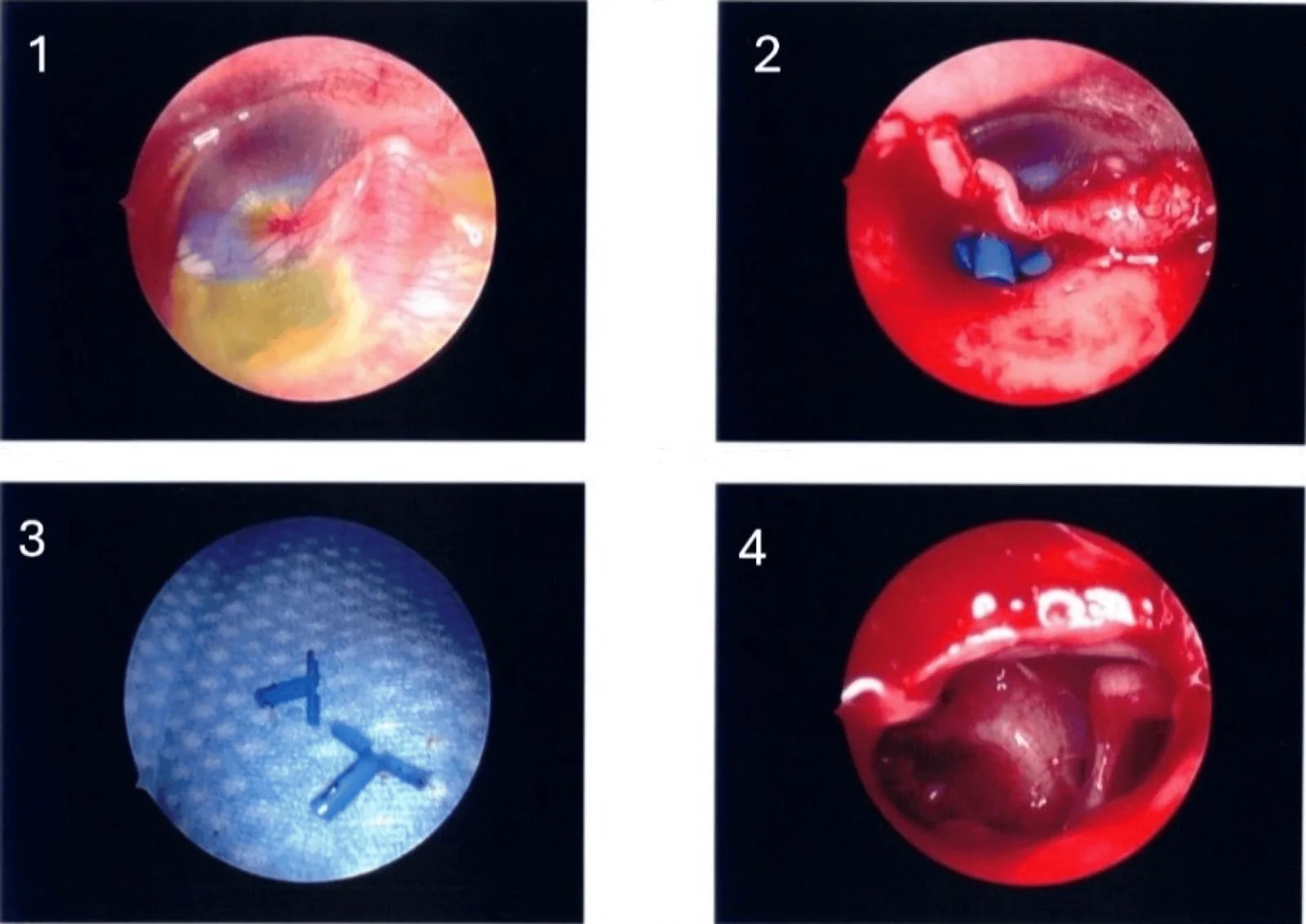
Panel 1 shows the left tympanic membrane with the medially displaced tympanostomy tubes behind the membrane. Panel 2 depicts the middle ear space after elevation of the tympanic membrane. Panel 3 demonstrates the two tubes following their removal from left middle ear space, and Panel 4 illustrates the ossicles visualized within the middle ear space.

The postoperative course was uncomplicated. The patient was maintained on dry ear precautions and had no subsequent episodes of otitis media, otorrhea, or otalgia. At six-month follow-up, she remained clinically stable without recurrent otologic symptoms. Importantly, her perceived hearing concerns had improved following allergy treatment prior to surgical removal of the medialized tubes. Postoperative audiometry is shown in Figure 3; these findings should therefore be interpreted in the context of her preoperative clinical improvement and should not be attributed solely to removal of the medialized tubes.

**Figure 3:**
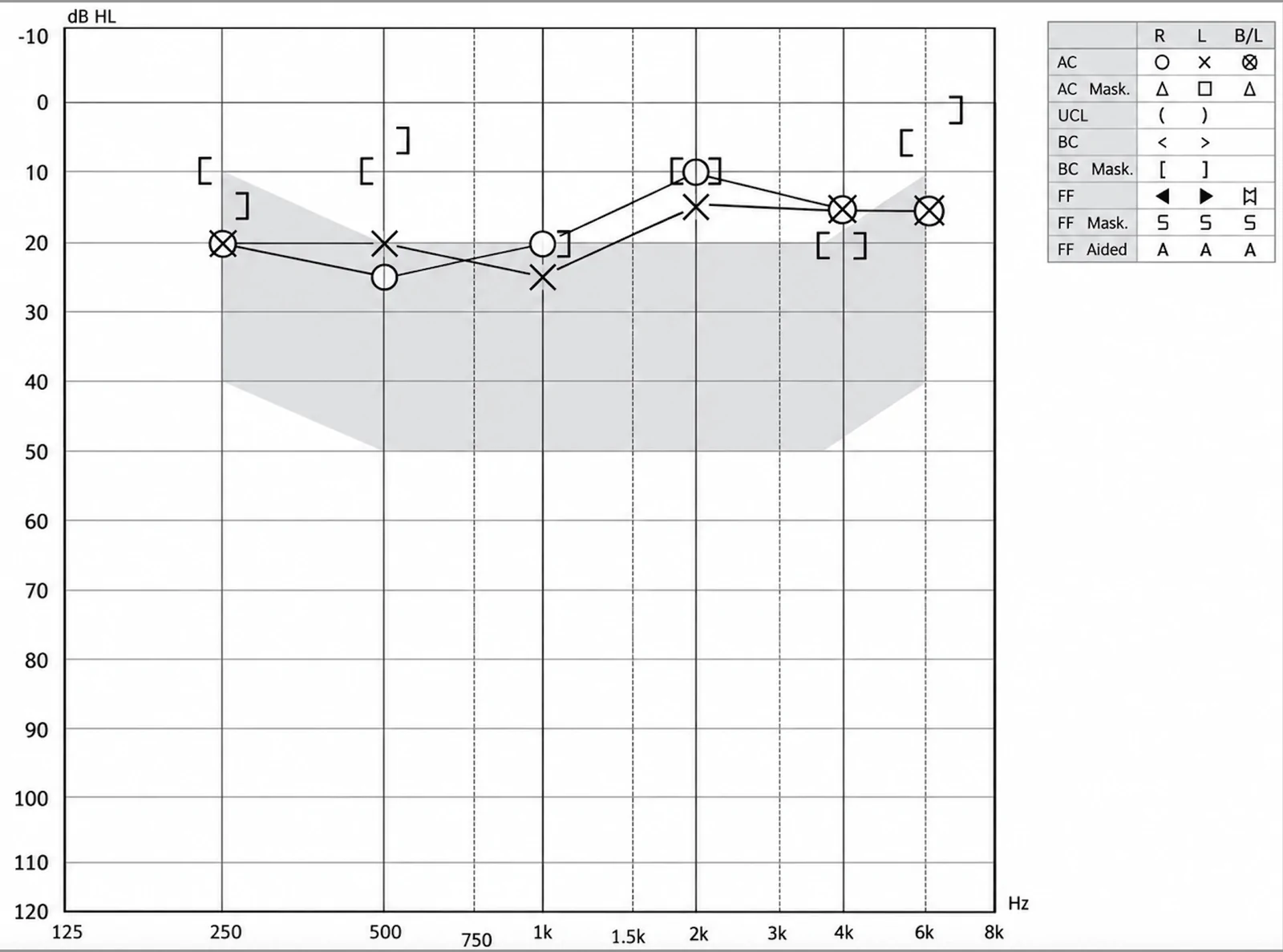
Postoperative pure-tone audiogram following surgical removal of the medialized tympanostomy tubes. Preoperative audiometry obtained from the referring practice demonstrated only a minimal conductive component. The patient’s perceived hearing concerns had improved following allergy treatment before surgical intervention; therefore, the postoperative audiometric findings should not be interpreted as evidence of hearing improvement attributable to removal of the medialized tubes.

## Discussion

Medialization of tympanostomy tubes into the middle ear space is an uncommon complication of tube placement. Although the mechanism is not always clear, displacement may occur during or after tube placement, with factors such as an excessively large myringotomy and persistent negative middle ear pressure proposed as potential contributors [1,2]. The clinical significance of a medialized tube varies, as some patients may remain asymptomatic while others may develop otalgia, aural fullness, conductive hearing loss, otorrhea, or inflammatory changes involving the middle ear.

Rare but more serious complications have also been described; a case report reported a perilymphatic fistula resulting from bony erosion caused by a medially displaced tympanostomy tube, highlighting the potential for retained medialized tubes to cause injury to adjacent middle ear structures [4]. Management of a medialized tympanostomy tube remains individualized. Observation may be reasonable in selected asymptomatic patients, whereas surgical removal may be considered when the tube is symptomatic, associated with middle-ear pathology, or located in a position that places adjacent structures at risk [3]. In the present case, the patient had remained free of otalgia and recurrent ear infections for six months following treatment of allergic rhinitis and associated ETD. Her perceived hearing concerns had also improved before surgical intervention. Therefore, postoperative clinical and audiometric findings should not be interpreted as evidence that removal of the medialized tubes directly resulted in improvement in hearing or otologic symptoms. Surgery was instead undertaken because of the persistent presence of the medialized tube and the potential risks associated with a retained foreign body within the middle ear [5].

The most notable feature of this case was the unexpected identification of two T-tubes within the middle ear space. The patient and available outside operative documentation both indicated placement of only one left-sided T-tube. She was therefore taken to the operating room with the expectation of removing a single medialized tube. The origin and timing of the second tube could not be established from the available history or records. Accordingly, this finding cannot be definitively characterized as recurrent or repeated medial migration.

No gross evidence of middle ear inflammation, ossicular injury, cholesteatoma, or other significant middle ear pathology was identified intraoperatively, and both medialized tubes were removed without complication. This finding supports the importance of careful otoscopic examination and review of prior operative documentation when evaluating patients with suspected medialized tympanostomy tubes, particularly when the available history or records may not fully account for the findings.

The patient's improvement following treatment of allergic rhinitis also highlights the potential contribution of nasal/allergic factors to her otologic symptoms. Although a causal relationship cannot be established from a single case, addressing contributing factors may be an important component of management in patients with tympanostomy tube complications.

Overall, this case adds to the limited adult literature describing medialized tympanostomy tubes and is notable for the unexpected presence of two tubes within the middle ear space. Because the origin of the second tube could not be determined, the finding should not be interpreted as definitive evidence of recurrent medial migration. Individualized assessment and appropriate follow-up remain important when a tympanostomy tube is identified within the middle ear.

## Conclusions

Medialization of tympanostomy tubes is a rare but recognized complication that may be associated with middle-ear inflammation, hearing loss, or injury to adjacent structures. This case highlights the unexpected identification of two medialized tympanostomy tubes in an adult with ETD, for which the origin and sequence of placement could not be definitively established. Both tubes were subsequently removed surgically without complication. Clinicians should remain aware of this uncommon complication and maintain appropriate otoscopic surveillance of patients with tympanostomy tubes to facilitate timely diagnosis and individualized management.
